# Consciousness: here, there and everywhere?

**DOI:** 10.1098/rstb.2014.0167

**Published:** 2015-05-19

**Authors:** Giulio Tononi, Christof Koch

**Affiliations:** 1 Department of Psychiatry, University of Wisconsin, Madison WI, USA; 2 Allen Institute for Brain Science, Seattle, WA, USA

**Keywords:** mind body problem, causation, existence, neuronal correlates of consciousness, awareness, cerebral cortex

## Abstract

The science of consciousness has made great strides by focusing on the behavioural and neuronal correlates of experience. However, while such correlates are important for progress to occur, they are not enough if we are to understand even basic facts, for example, why the cerebral cortex gives rise to consciousness but the cerebellum does not, though it has even more neurons and appears to be just as complicated. Moreover, correlates are of little help in many instances where we would like to know if consciousness is present: patients with a few remaining islands of functioning cortex, preterm infants, non-mammalian species and machines that are rapidly outperforming people at driving, recognizing faces and objects, and answering difficult questions. To address these issues, we need not only more data but also a theory of consciousness—one that says what experience is and what type of physical systems can have it. Integrated information theory (IIT) does so by starting from experience itself via five phenomenological axioms: *intrinsic existence, composition, information, integration* and *exclusion*. From these it derives five postulates about the properties required of physical mechanisms to support consciousness. The theory provides a principled account of both the quantity and the quality of an individual experience (a quale), and a calculus to evaluate whether or not a particular physical system is conscious and of what. Moreover, IIT can explain a range of clinical and laboratory findings, makes a number of testable predictions and extrapolates to a number of problematic conditions. The theory holds that consciousness is a fundamental property possessed by physical systems having specific causal properties. It predicts that consciousness is graded, is common among biological organisms and can occur in some very simple systems. Conversely, it predicts that feed-forward networks, even complex ones, are not conscious, nor are aggregates such as groups of individuals or heaps of sand. Also, in sharp contrast to widespread functionalist beliefs, IIT implies that digital computers, even if their behaviour were to be functionally equivalent to ours, and even if they were to run faithful simulations of the human brain, would experience next to nothing.

## 1. Consciousness: here, there and everywhere?

I know I am conscious: I am seeing, hearing, feeling something *here*, inside my own head. But is consciousness—subjective experience—also *there,* not only in other people's heads, but also in the head of animals? And perhaps *everywhere,* pervading the cosmos, as in old panpsychist traditions and in the Beatles' song? While these kinds of questions may seem scientifically inappropriate, we argue below that they can be approached in a principled and testable manner. Moreover, obtaining an answer is urgent, not only because of difficult clinical cases and in our interactions with other species but also because of the advent of machines that are getting closer to passing the Turing test—computers programmed to perform many tasks as well as us, and often far better than some brain-damaged patients.

## 2. Here

That I am conscious, here and now, is the one fact I am absolutely certain of—all the rest is conjecture. This is, of course, the gist of the most famous deduction in Western thought, Descartes' *je pense, donc je suis*. Everything else—what I think I know about my body, about other people, dogs, trees, mountains and stars, is inferential. It is a reasonable inference, corroborated first by the beliefs of my fellow humans and then by the intersubjective methods of science. Yet consciousness itself—the central fact of existence—still demands a rational explanation.

The past two centuries of clinical and laboratory studies have revealed an intimate relationship between the conscious mind and the brain, but the exact nature of this relationship remains elusive. Why is the brain associated with consciousness but not the liver or the heart, as previous cultures believed? Why certain parts of the brain and not others? Why is consciousness lost in some stages of sleep? Why does red feel like red and not like the sound of a violin? Is consciousness just an epiphenomenon, or does it have a function? Can computers be conscious? Could a system behave like us and yet be devoid of consciousness—a zombie? Such questions seem to resist the empirical, reductionist approach that has been so successful for other aspects of the natural world. Nevertheless, thanks to experimental and theoretical progress in the past decades [1–5], we are in a better position to understand which systems under which conditions can be conscious. That is, the study of consciousness is becoming a science. In doing so, it is leaving behind the defeatist dictum of the physiologist Emil du Bois-Reymond, *ignoramus et ignorabimus* (we don't know and never will), espousing instead the upbeat maxim of the mathematician David Hilbert, *Wir müssen wissen*—*wir werden wissen* (we must know and we will know).

## 3. There

We usually grant consciousness to others—of the same kind we experience in the privacy of our own mind—if they can tell us what they feel, or if they look and behave more or less like us. However, we become less and less confident in attributing consciousness to those who cannot talk about their experiences, such as infants or severely brain injured patients. Many assume that animals closely related to *homo sapiens*—apes and other primates—are conscious, though presumably less than we are, based on the similarity of their behaviour and their brain. But should we attribute experience to all mammals,^1^ to all vertebrates, to invertebrates such as cephalopods and bees or even to all multi-cellular animals? What about cultured organoids that mimic the cellular organization of the developing human brain [8]? And finally, what about the sophisticated machines that run software designed to substitute for conscious humans in many complicated tasks?

### (a) Behavioural correlates of consciousness and reportability

**Figure 1.**
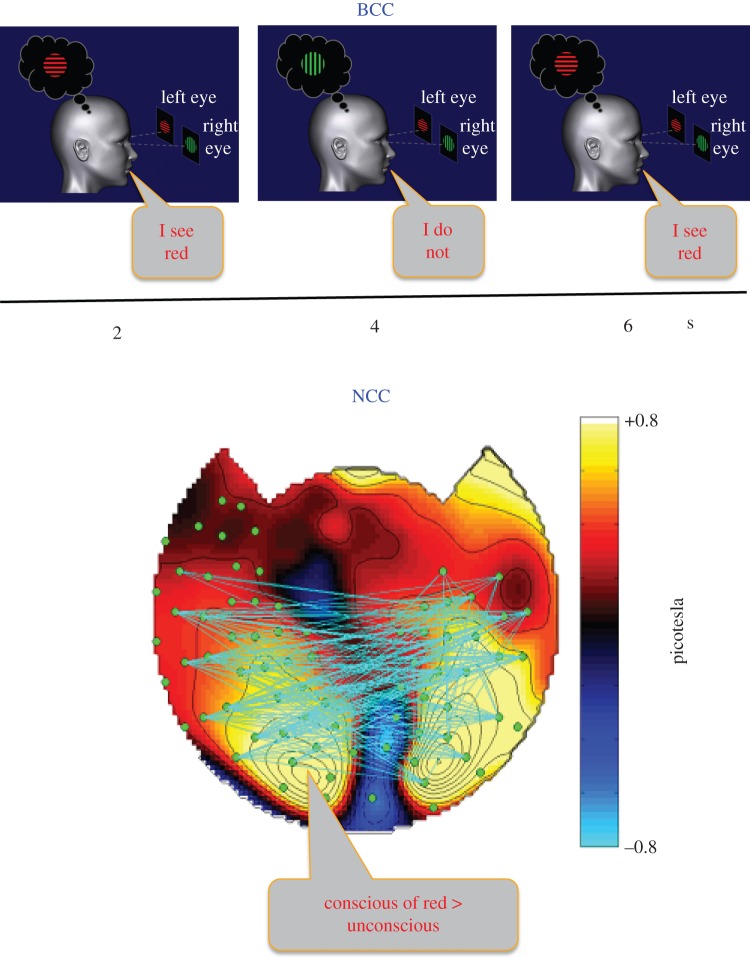
Behavioural (BCC) and neuronal correlates of consciousness (NCC). The top row shows a schematic diagram of a binocular rivalry experiment. A horizontal red grating is shown to the left eye and a vertical green grating to the right eye throughout the experiment (courtesy of Naotsugu Tsuchiya and Olivia Carter). The subject does not see a juxtaposition of both stimuli but experiences either the red grating or the green one, switching back and forth every few seconds. Even if the stimuli do not change, what one sees consciously does, as is inferred by the subject's report. The bottom row shows the results of an experiment using magnetoencephalography (MEG), in which the red grating was flashed at one frequency and the green one at another. Yellow indicates areas of the cortex (seen from the top) that had more power at the frequency of the red grating when it was experienced than when it was not. The cyan lines indicate increased coherence (synchronization) between distant brain regions associated with experiencing the grating (from [9]).

Traditionally, we assess consciousness by observing behaviour (figure 1*a*). If someone is awake and acts meaningfully, we have little doubt he is conscious. If he speaks, and especially if he can answer questions about what he is conscious of, we are fully confident. In the laboratory, the ability to report one's experiences has become the gold standard for judging the presence of consciousness. Reportability is often reduced to a binary forced choice, in which the subject pushes one of two buttons for ‘seen’ versus ‘not seen’, or ‘angry face’ versus ‘happy face’. One can also ask subjects how confident they are in their judgements (*confidence rating* [10]), ask them to further describe their experiences (*perceptual awareness scale* [11,12]) or get them to make an economic judgement following each response (*post-decision wagering* [13]). These kinds of meta-cognitive and confidence reports can also be obtained from trained monkeys and other animals, with so many similarities to our own reports that there is little doubt as to the presence of consciousness [14,15] (but see [16]).

But behaviour can be misleading: a person may walk and speak in her sleep, yet it is quite dubious whether she is experiencing anything. Or a person can be asleep, immobile, silent and unresponsive, yet she may be dreaming—vividly conscious of an imaginary environment. In such cases, reportability can be used as retrospective evidence of consciousness, by waking up the sleeper to obtain a ‘dream report’. However, reportability, too, can be problematic. Since we obviously experience things in dreams whether or not we are woken up to report them, we should accept the possibility that in certain situations consciousness can be present even if it is not reported [17,18]. Moreover, insisting on reportability elevates language to a king-maker role, which makes inferring consciousness in non-verbal infants, preterm babies, fetuses or animals problematic.^2^ Clearly, if we want to understand what is really going on, we must also investigate the brain mechanisms that underlie consciousness.

### (b) Neural correlates of consciousness

The neural correlates of consciousness (NCC) have been defined as the minimal neural mechanisms that are jointly sufficient for any one conscious percept, thought or memory, under constant background conditions (figure 1*b*) [1,23,24]. The latter are the distal or proximal *enabling factors* that must be present for any conscious experience to occur—the heart must beat and supply the brain with oxygenated blood, various nuclei in the midbrain reticular formation and brainstem must be active [25–27], cholinergic release needs to occur within the cortico-thalamic complex [28] and so on.

Every experience will have an associated NCC: one for seeing a red patch, another one for hearing a high C. Inducing the NCC by manipulating the relevant neuronal populations via magnetic stimulation, optogenetics or other means will give rise to the associated conscious percept. Interfering with the NCC by disabling the underlying neural circuits will eliminate the percept.

**Figure 2.**
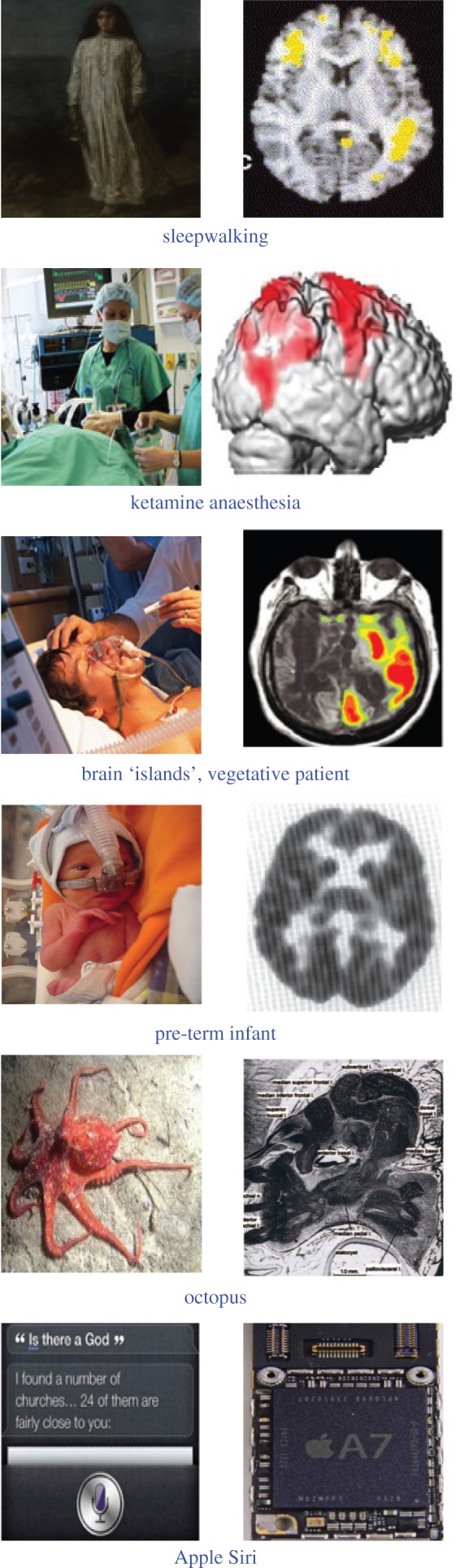
Six instances in which it becomes progressively more difficult to infer the existence of consciousness, since the behavioural repertoire and the underlying mechanisms (brains) differ substantially from that of typical persons able to speak about their experiences (figure 1).

The NCC are typically assessed by determining which aspects of neural function change depending on whether a subject is conscious or not, as established using behavioural reports. This can be done by considering a global change in the level of consciousness, as when awareness is lost during deep sleep or general anaesthesia [29,30]. Or it can be done by considering changes in a particular content of consciousness, as when a subject's awareness of a particular stimulus is experimentally manipulated (‘seen’ versus ‘not seen’ [31,32]). In optimally controlled experiments, the stimulus and the behavioural report (such as a button press) are kept constant while the subject sometimes sees the percept and sometimes does not [3,33,34]. Once a particular NCC has been sufficiently validated, it can be used to extrapolate to situations in which reports are not available. Both functional brain imaging in magnetic scanners and as high-density electroencephalography (EEG) recordings from outside the skull have been put to use to track down the footprints of consciousness in the brain of healthy adult observers. Popular candidates include strong activation of high level fronto-parietal cortices (figure 1*b*), high-frequency electrical activity in the gamma range (35–80 Hz), and the occurrence of an EEG event known as the P300 wave [1,3,29]. However, there is still no consensus on whether any of these signs can be treated as reliable ‘signatures’ of consciousness. In particular, there can be consciousness without frontal cortex involvement [35–37], gamma activity without consciousness [38], such as during anaesthesia [39,40], and consciousness without a frontal P300, for example, during dreaming sleep [41,42]. Moreover, it is likely that many of the signatures proposed as possible NCC may actually be correlates of neural activity that is needed leading up to a conscious percept [43,44], or for giving a report following a conscious percept [36,37,44], rather than for having an experience. A major challenge is to keep constant cognitive functions such as selective attention, memory, decision making and task monitoring, in order to isolate the ‘naked’ substrate of consciousness at the neuronal level [45,46]. Finally, NCC obtained in healthy adults may or may not apply to brain-damaged patients, to infants, to animals very different from us, not to mention machines (figure 2).

### (c) Patients and infants

Patients with widespread cortical or thalamic damage pose a poignant challenge. Emergency room personnel quickly evaluate the severity of a head injury behaviourally by assigning a number to a patient's auditory, visual, verbal and motor functions as well as communication and arousal level. Various NCC, such as the presence of a P300 wave in response to a non-standard stimulus, are increasingly being used to complement the behavioural assessment and occasionally modify the diagnosis. In some cases, NCC can be decisive. Thus, if a patient who lies mute and immobile can nevertheless respond to commands by appropriately activating certain brain areas, it is fair to conclude that she is conscious [47]. Yet most of the proposed ‘signatures’ of consciousness are inadequate. For example, the P300 wave is absent in many minimally conscious patients and even in some brain-damaged patients who can communicate [48]. And what should one make of patients in whom, amidst widespread destruction and inactivity, one or a few isolated cortical areas may show signs of metabolic activation and electrophysiological ‘markers’ of consciousness [49]? Is an island of functioning brain tissue sufficient for generating a limited kind of awareness, maybe just awareness of sound or of pain? In other words, ‘what is it like’ to be a brain island, if it feels like anything at all? And how big must the island be to qualify?

By the same token, what is it like to be a newborn baby with an immature brain and restricted connectivity among cortical structures [50]? Again, considering NCC can be helpful: for example, a wave resembling the P300 wave has been reported in six to 16 months old infants, although weaker, more variable and delayed compared with adults [51]. But does this mean that newborn and preterm babies or even fetuses experience nothing because they do not show a P300?

### (d) Animals

The problem becomes even more acute when turning to other species. The study of consciousness in nature has been hindered for centuries by a strong belief in human exceptionalism. Yet the range and complexity of animal behaviour has laid rest to this belief, at least among biologists. This is particularly true for mammals. In psychophysical tasks involving simple button presses, trained macaque monkeys act very similarly to human volunteers, including signalling when they do not see anything [14]. Visual recognition of self, meta-cognition (knowing one's mind), theory of mind, empathy and long-range planning have all been demonstrated in primates, rodents and other orders [52].

It is also difficult to find anything exceptional about the human brain [53]. Its constitutive genes, synapses, neurons and other cells are similar to those found in many other species. Even its size is not so special, as elephants, dolphins and whales have even bigger brains [54]. Only an expert neuroanatomist, armed with a microscope, can tell a grain-sized piece of neocortex of a mouse from that of a monkey or a human. Biologists emphasize this structural and behavioural continuity by distinguishing between *non-human* and *human* animals [55]*.* Given this continuity, it seems unjustified to claim that only one species has consciousness while everybody else is devoid of experience, is a zombie. It is far more likely that all mammals have at least some conscious experiences, can hear the sounds and see the sights of life.

As we consider species that are progressively further removed from *Homo sapiens* in evolutionary and neuronal terms, the case for consciousness becomes more difficult to make. Two observations, one relating to complexity of behaviour and another one to complexity of the underlying nervous system, are critical. First, ravens, crows, magpies, parrots and other birds, tuna, coelacanths and other fish, octopuses and other cephalopods, bees and other members of the vast class of insects are all capable of sophisticated, learnt, non-stereotyped behaviours that we associate with consciousness if carried out by people [56–58]. Darwin himself set out ‘to learn how far the worms acted consciously’ and concluded that there was no absolute threshold between ‘lower’ and ‘higher’ animals, including humans, which would assign higher mental powers to one but not to the other [59]. Second, the nervous systems of these species display a vast and ill-understood complexity. The bee contains about 800 000 nerve cells whose morphological and electrical heterogeneity rivals that of any neocortical neuron. These cells are assembled in highly nonlinear feedback circuits whose density is up to ten times higher than that of neocortex [60]. Thus, neural signatures of consciousness that have some validity in humans and other mammals may not apply at all to invertebrates.

On the other hand, the lessons learnt from studying the behavioural (BCC) and neuronal correlates of consciousness in people must make us cautious about inferring its presence in creatures very different from us, no matter how sophisticated their behaviour and how complicated their brain. Humans can perform complex behaviours—recognizing whether a scene is congruous or incongruous, controlling the size, orientation and strength of how one's finger should grip an object, doing simple arithmetic, detecting the meaning of words or rapid keyboard typing—in a seemingly non-conscious manner [61–66]. When a bee navigates a maze, does it do so like when we consciously deliberate whether to turn right or left, or rather like when we type on a keyboard? Similarly, consider that an extraordinarily complicated neuronal structure in our brain, the cerebellum, home to 69 of the 86 billion nerve cells that make up the human brain [54], apparently has little to do with consciousness. Patients who lose part or nearly all of their cerebellum owing to stroke or other trauma show ataxia, slurred speech and unsteady gait [67] but do not complain of a loss or diminution of consciousness. Is the bee's brain central complex more like the cerebellum or more like the cerebral cortex with respect to experience? Thus, the extent to which non-mammalian species share with us the gift of subjective experience remains hard to fathom.^3^

### (e) Machines

Difficulties in attributing sentience become even more apparent when considering digital computers. These have a radically different architecture and provenance from biological organisms shaped by natural selection. Owing to the relentless decrease in transistor size over the past 50 years and the concomitant exponential increase in computational power and memory capacity, present-day computers executing appropriate algorithms outperform us in many tasks that were thought to be the sole prerogative of the human mind. Prominent examples include IBM's Deep Blue that beat the reigning chess world master in 1997; another IBM computer, Watson, that can answer questions posed in spoken English and won the quiz show *Jeopardy* in 2011; smart phones that answer questions by speech; Google's driverless cars that have logged more than half a million miles on open roads; and machine vision algorithms for face detection in security and commercial applications [68]. People playing chess, supplying meaningful answers to questions, driving a car or picking out a face are assumed to be conscious. But should we say the same for these digital creatures?

## 4. Integrated information theory

Clearly, as we move away from people, BCC and NCC become progressively less helpful to establish the presence of consciousness. Even in the normal human brain, we need to understand *why* and *how* certain structures are associated with experience (the cerebral cortex or, possibly, the claustrum [69,70]) while others are not (the cerebellum), and why they do so under certain conditions (wake, dreams) and not others (deep sleep, seizures). Some philosophers have claimed that the problem of explaining how matter can give rise to consciousness may forever elude us, dubbing it the *Hard* problem [71–73]. Indeed, as long as one starts from the brain and asks how it could possibly give rise to experience—in effect trying to ‘distill’ mind out of matter [74], the problem may be not only hard, but almost impossible to solve. But things may be less hard if one takes the opposite approach: start from consciousness itself, by identifying its essential properties, and then ask what kinds of physical mechanisms could possibly account for them. This is the approach taken by integrated information theory (IIT) [75–79], an evolving formal and quantitative framework that provides a principled account for what it takes for consciousness to arise, offers a parsimonious explanation for the empirical evidence, makes testable predictions and permits inferences and extrapolations (table 1).^4^

**Table 1.** Some terms used in integrated information theory (IIT).

**Figure 3.**
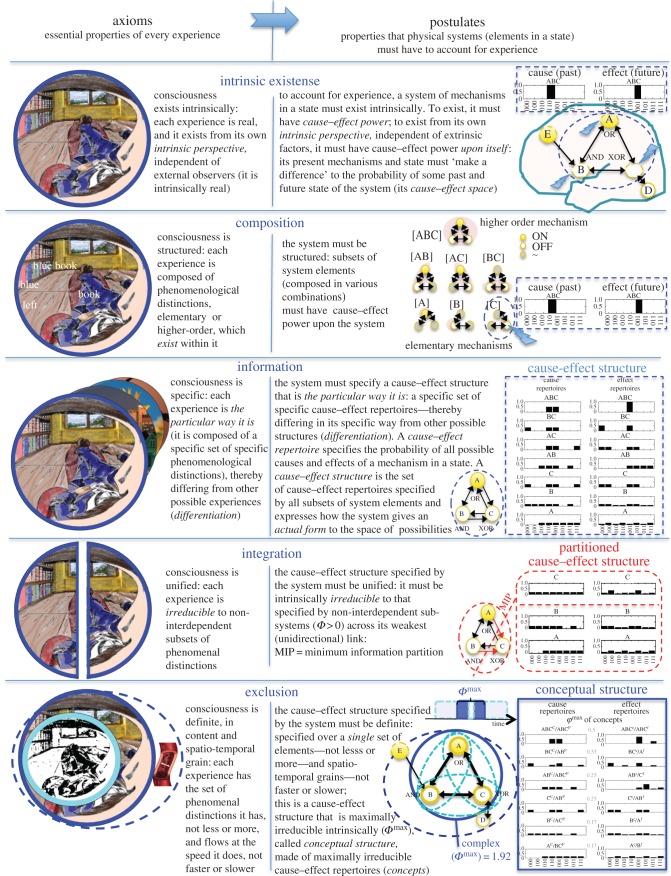
Axioms and postulates of integrated information theory (IIT). The illustration is a colourized version of Ernst Mach's ‘View from the left eye’ [84]. See also the mechanisms in figure 4.

*Axioms*. Properties of consciousness that are taken as self-evident. The only truths that, with Descartes, cannot be doubted and do not need proof. They are intrinsic existence, composition, information, integration and exclusion (figure 3, left).
*Postulates*. Assumptions, derived from axioms, about the physical substrates of consciousness (mechanisms must have cause–effect power, be irreducible, etc.), which can be formalized and form the basis of the mathematical framework of IIT. It is as yet unproven whether the mapping from axioms to postulates is unique. There are five postulates, matching the five axioms (figure 3, right).
*Element*. A minimal component of a system, for example, a neuron in the brain or a logic gate in a computer, having at least two states, inputs that can affect those states and outputs that depend on them. Strictly speaking, such elements are *macro*-elements constituted of *micro*-elements such as molecules, which are constituted in turn of atoms and so on. IIT predicts that, if neurons are the relevant elements for consciousness, intrinsic cause–effect power within the system must be highest at the level of such macro-elements rather than at the level of the constituting micro-elements [79].
*Mechanism*. Any subset of elements within a system, first- and higher order, including the system itself, which has cause–effect power within the system.
*Cause–effect repertoire*. The probability distribution of potential past and future states of a system as informed by a mechanism in its current state.
*Integrated information* (*φ*). Information that is specified by a mechanism above and beyond the information specified by its (minimal) parts. *φ* measures the integration or irreducibility of the cause–effect repertoire specified by a mechanism.
*MIP* (*minimum information partition*). The partition that makes the least difference—in other words, the minimum ‘difference’ partition.
*Complex*. A set of elements within a system that specifies a local maximum of integrated conceptual information *Φ*^max^. Only a complex exists as an entity from its own intrinsic perspective.
*Concept*. A mechanism and the maximally irreducible cause–effect repertoire it specifies, with its associated value of integrated information *φ*^max^. The concept expresses the cause–effect power of a mechanism within a complex.
*Conceptual structure*. The set of all concepts specified by a system set with their respective *φ*^max^ values, which can be plotted as a constellation of concepts in cause–effect space.
*Cause–effect space* (*or qualia space*). A high-dimensional space with one axis for each possible past and future state of the system in which a conceptual structure can be represented.
*Integrated conceptual information* (*Φ*). Conceptual information that is specified by a system above and beyond the conceptual information specified by its (minimal) parts. *Φ* measures the intrinsic integration or irreducibility of a constellation of concepts (integration at the system level), a non-negative number.
*Quale*. A conceptual structure specified by a complex in a state that is maximally irreducible intrinsically (synonymous with constellation in qualia space).

### (a) Axioms: essential phenomenological properties of consciousness

Taking consciousness as primary, IIT first identifies *axioms* of experience (figure 3, left), then derives a set of corresponding *postulates* (figure 3, right) about its physical substrate [77,80]. The axioms of IIT are assumptions about our own experience that are the starting point for the theory. Ideally, axioms are essential (apply to all experiences), complete (include all the essential properties shared by every experience), consistent (lack contradictions) and independent (not derivable from each other). Whether the current set of five axioms are truly valid, complete and independent remains open.^5^ The five axioms are intrinsic existence, composition, information, integration and exclusion.

#### (i) Intrinsic existence

Consciousness *exists*: my experience just *is*. Indeed, that my experience here and now exists—it is real or actual—is the only fact I am immediately and absolutely sure of, as Descartes realized four centuries ago. Moreover, my experience exists from its own *intrinsic* perspective, independent of external observers.

#### (ii) Composition

Consciousness is *structured*: each experience is composed of many *phenomenological distinctions*, elementary or higher order, which also exist. Within the same experience, for example, I may distinguish a book, a blue colour, a blue book and so on.

#### (iii) Information

Consciousness is *specific*: each experience is *the particular way it is*—it is composed of a specific set of specific phenomenal distinctions—thereby differing from other possible experiences (*differentiation*). Thus, an experience of pure darkness and silence is what it is because, among other things, it is not filled with light and sound, colours and shapes, there are no books, no blue books and so on. And being that way, it necessarily differs from a large number of alternative experiences I could have. Just consider all the frames of all possible movies: the associated visual percepts are but a small subset of all possible experiences.

#### (iv) Integration

Consciousness is *unified*: each experience is *irreducible* to non-interdependent subsets of phenomenal distinctions. Thus, I experience a whole visual scene, not the left side of the visual field independent of the right side (and vice versa). For example, the experience of seeing written in the middle of a blank page the word ‘HONEYMOON’ is irreducible to an experience of seeing ‘HONEY’ on the left plus the experience of seeing ‘MOON’ on the right. Similarly, seeing a blue book is irreducible to seeing a grey book plus the disembodied colour blue.

#### (v) Exclusion

Consciousness is *definite*, in content and spatio-temporal grain: each experience has the set of phenomenal distinctions it has, neither less (a subset) nor more (a superset), and it flows at the speed it flows, neither faster nor slower. Thus, the experience I am having is of seeing a body on a bed in a bedroom, a bookcase with books, one of which is a blue book, but I am not having an experience with less content—say, one lacking the phenomenal distinction blue/not blue, or coloured/not coloured; nor am I having an experience with more content—say, one endowed with the additional phenomenal distinction high/low blood pressure. Similarly, my experience flows at a particular speed—each experience encompassing a hundred milliseconds or so—but I am not having experience that encompasses just a few milliseconds or instead minutes or hours.

### (b) Postulates: properties that physical mechanisms must have to support consciousness

To parallel these axioms that capture the essential properties of every experience, IIT proposes a set of postulates concerning the requirements that must be satisfied by any physical system to account for experience (figure 3, right). For simplicity, physical systems are considered as *elements in a state*, such as neurons or logic gates that are either ON or OFF. All that is required is that such elements have two or more internal states, inputs that can influence these states in a certain way and outputs that in turn depend on these states.

#### (i) Intrinsic existence

A system of mechanisms in a state must *exist intrinsically*. Specifically, in order to exist, it must have *cause–effect power*, as there is no point in assuming that something exists if nothing can make a difference to it, or if it cannot make a difference to anything [88].^6^ Moreover, to exist from its own intrinsic perspective, independent of external observers, it must have cause–effect power *upon* itself, independent of extrinsic factors (figure 3, intrinsic existence). Cause–effect power can be established by considering a *cause–effect space* with an axis for every possible state of the system in the past (causes) and in the future (effects). Within this space, it is enough to show that an ‘intervention’ that sets the system in some initial state, keeping the state of the elements outside the system fixed (background conditions), can lead with probability different from chance to its present state (cause); conversely, setting the system to its present state leads with probability different from chance to some other state (effect).

#### (ii) Composition

The system must be *structured*: subsets of the elementary mechanisms of the system, *composed* in various combinations, also have cause–effect power within the system. Thus, if a system ABC comprises elements A, B and C (figure 3, composition), any subset of elements, including A, B, C; AB, AC, BC; as well as the entire system, ABC, can compose a mechanism having cause–effect power. Composition allows for elementary (first-order) mechanisms to form distinct higher order mechanisms, and for multiple mechanisms to form a structure.

#### (iii) Information

The system must *specify* a cause–effect structure that is *the particular way it is*: a specific set of specific cause–effect repertoires—thereby differing from other possible ones (*differentiation*). A *cause–effect repertoire* characterizes in full the cause–effect power of a mechanism within a system by making explicit all its cause–effect properties. It can be determined by perturbing the system in all possible ways to assess how a mechanism in its present state makes a difference to the probability of the past and future states of the system. Together, the cause–effect repertoires specified by each composition of elements within a system specify a *cause–effect structure*. Consider for example, within the system ABC (figure 3, information), the mechanism implemented by element C, an XOR gate with two inputs (A and B) and two outputs (the OR gate A and the AND gate B). If C is OFF, its cause repertoire specifies that, at the previous time step, A and B must have been either in the state OFF,OFF or in the state ON,ON, rather than in the other two possible states (OFF,ON; ON,OFF); and its effect repertoire specifies that the next time step B will have to be OFF, rather than ON. Its cause–effect repertoire is specific: it would be different if the state of C were different (ON), or if C were a different mechanism (say, an AND gate). Similar considerations apply to every other mechanism of the system, implemented by different compositions of elements. Thus, the cause–effect repertoire specifies the full cause–effect power of a mechanism in a particular state, and the cause–effect structure specifies the full cause–effect power of a system of mechanisms. Note that the notion of information in IIT differs substantially from that in communication theory or in common language, but it is faithful to its etymology: information refers to how a system of mechanisms in a state, through its cause–effect power, specifies a form (‘informs’ a conceptual structure) in the space of possibilities.

#### (iv) Integration

The cause–effect structure specified by the system must be *unified*: it must be intrinsically *irreducible* to that specified by non-interdependent sub-systems obtained by unidirectional partitions. Partitions are taken unidirectionally to ensure that cause–effect power is intrinsically irreducible—from the system's intrinsic perspective—which implies that every part of the system must be able to both affect and be affected by the rest of the system. Intrinsic irreducibility can be measured as integrated information (‘big phi’ or *Φ*, a non-negative number), which quantifies to what extent the cause–effect structure specified by a system's mechanisms changes if the system is partitioned (cut or reduced) along its minimum partition (the one that makes the least difference). For example, the system in figure 3 is integrated, because partitioning it through its weakest link destroys several cause–effect repertoires and changes others (compare the cause–effect structure under ‘information’ and under ‘integration’ in figure 3). By contrast, if a system of mechanisms can be divided into two sub-systems and the partition makes no difference to the associated cause–effect structure, then the whole is reducible to those parts. Being intrinsically irreducible is another precondition for existence having to do with causation: there is no point in assuming that the whole exists in and of itself, if it has no cause–effect power above and beyond its parts. This postulate also applies to individual mechanisms: a subset of elements can contribute a specific aspect of experience only if its cause–effect repertoire within the system is irreducible by the minimum partition of the mechanism (‘small phi’ or *φ*).

#### (v) Exclusion

The cause–effect structure specified by the system must be *definite*: it is specified over a *single* set of elements—neither less nor more—the one over which it is *maximally irreducible* (*Φ*^max^) from its intrinsic perspective, thus laying maximal claim to existence. For example (figure 3, exclusion), within ABCDE, many candidate systems could specify cause–effect structures, including AB, AC, BC, ABC, ABCD, ABCDE and so on. Among these, the system that specifies the cause–effect structure that is maximally irreducible intrinsically is the set of elements ABC, rather than any of its subsets or supersets. The exclusion postulate provides a sufficient reason why the contents of the experience should be what they are—neither less nor more. With respect to causation, this has the consequence that the ‘winning’ cause–effect structure excludes alternative cause–effect structures specified over overlapping elements: if a mechanism in a state (say A OFF) specifies a particular cause–effect repertoire within one system (ABC), it should not *additionally* specify an overlapping cause–effect repertoire as part of other, overlapping systems (say AB or ABCD), otherwise one would be counting multiple times the difference that mechanism makes. The exclusion postulate can be said to enforce Occam's razor (entities should not be multiplied beyond necessity): it is more parsimonious to postulate the existence of a single cause–effect structure over a system of elements—the one that is maximally irreducible—than a multitude of overlapping cause–effect structures whose existence would make no further difference. The exclusion postulate also applies to individual mechanisms: a subset of elements in a state specifies the cause–effect repertoire within the system that is maximally irreducible (*φ*^max^), called a *core concept*, or *concept* for short. Again, it cannot *additionally* specify a cause–effect repertoire overlapping over the same elements, because otherwise the difference a mechanism makes would be counted multiple times. A maximally irreducible cause–effect structure composed of concepts is called a *conceptual structure*. The system of mechanisms that specifies a conceptual structure is called a *complex.*^7^ It is useful to think of a conceptual structure as existing as a form in cause–effect space, whose axes are given by all possible past and future states of the complex. In this space, every concept is a point (star), whose size is given by its irreducibility *φ*^max^, and a conceptual structure is a ‘constellation’ of points, that is, a *form*. Finally, this postulate also applies to spatio-temporal grain. For example, a mechanism cannot have effects at a fine temporal grain, and additional effects at a coarser grain, otherwise causal exclusion would be violated. On the other hand, if the effects at a coarser grain are more irreducible than those at a finer grain, then the coarser grain of causation excludes the finer one [79].^8^

### (c) The central identity: experience as a conceptual structure

**Figure 4.**
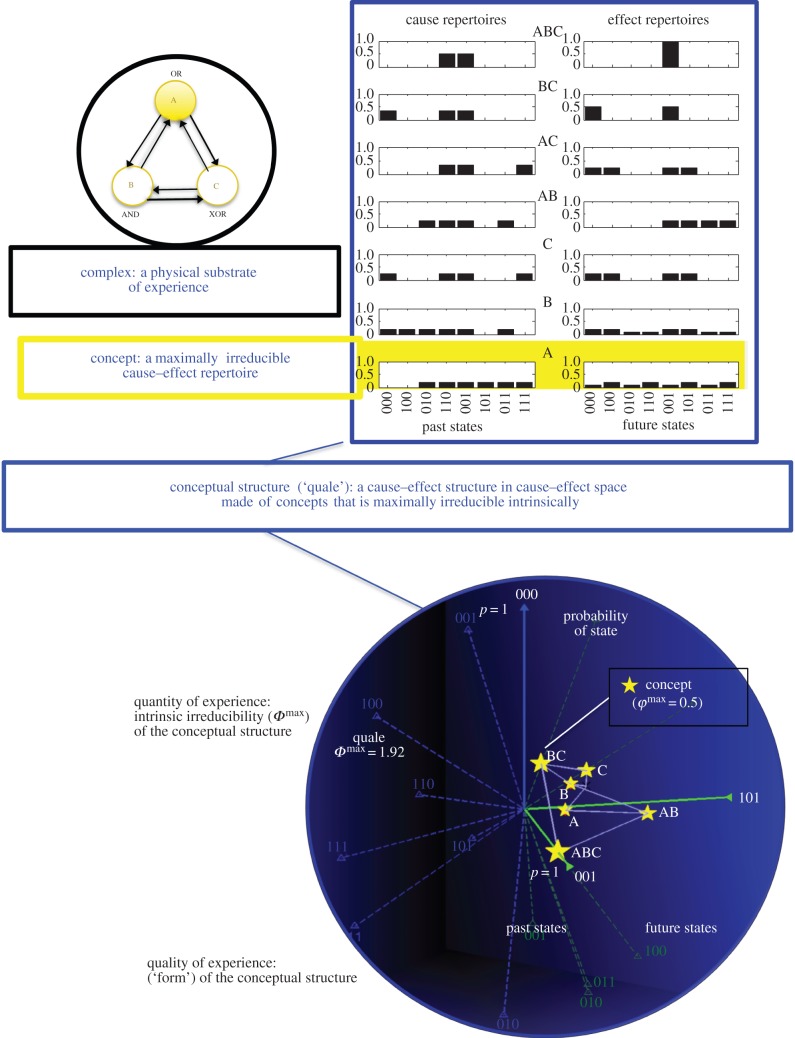
A didactic example of how to calculate the quality and quantity of consciousness given a system of elements in a state. On the upper left are three gates with binary states (either ON or OFF: ABC = 100; see also figure 3) that are wired together as shown. An analysis based on the postulates of IIT [80] reveals that the system forms a complex. The complex in its present state specifies a quale—a conceptual structure that is maximally irreducible intrinsically. The quale is presented both as the set of maximally irreducible cause–effect repertoires (concepts) specified by each mechanism (top) and as a two-dimensional projection in which each concept is a ‘star’ in cause–effect space (bottom). Cause–effect space or qualia space is a high-dimensional (here, 2 × 8 dimensions) space in which each axis is a possible past (in blue) and future (in green) state of the complex, and the position along the axis is the probability of that state. Each concept is a star whose position indicates how a mechanism composed of a subset of elements affects the probability of past and future states of the system (its cause–effect repertoire, which specifies what the concept contributes to experience) and whose size (*φ*^max^) measures how irreducible the concept is (how much it contributes to experience). In IIT, *Φ*^max^—a non-negative number—measures the intrinsic irreducibility of the entire quale, how much consciousness there is—the quantity of experience. The ‘form’ or shape of the quale (constellation of stars) is identical to the quality of the experience. Different shapes correspond to different experiences: they feel the way they do—red feeling different from blue or from a headache—because of the distinct shapes of their qualia.

Altogether, the elements of a complex in a state, composed into higher order mechanisms that specify concepts, form a *conceptual structure* that is *maximally irreducible intrinsically*, also known as a *quale*. The constellation of all concepts specifies the overall *form* or shape of the quale (figure 4).

On this basis, the central identity of IIT can be formulated quite simply: *an experience is identical to a conceptual structure* that is *maximally irreducible intrinsically*. More precisely, a conceptual structure completely specifies both the quantity and the quality of experience: *how much* the system exists—the quantity or level of consciousness—is measured by its *Φ*^max^ value—the intrinsic irreducibility of the conceptual structure; *which way* it exists—the quality or content of consciousness—is specified by the shape of the conceptual structure. If a system has *Φ*^max^ = 0, meaning that its cause–effect power is completely reducible to that of its parts, it cannot lay claim to existing. If *Φ*^max^ > 0, the system cannot be reduced to its parts, so it exists in and of itself. More generally, the larger *Φ*^max^, the more a system can lay claim to existing in a fuller sense than systems with lower *Φ*^max^. According to IIT, the quantity and quality of an experience are an intrinsic, fundamental property of a complex of mechanisms in a state—the property of informing or shaping the space of possibilities (past and future states) in a particular way, just as it is considered to be intrinsic to a mass to bend space–time around it.^9^

At any given time, then, consciousness is supported by a set of neuronal elements forming a complex of high *Φ*^max^ that specifies a conceptual structure that is maximally irreducible intrinsically. The particular set of neurons that form the major complex, the one of highest *Φ*^max^ in the brain, may change to some extent from moment to moment, as well as their state—which neurons are firing and which are not. For example, let us assume that while I watch a scene of a movie containing the actress Jennifer Aniston (JA), the major complex in my brain is made up of neurons within certain parts of the cerebral cortex.^10^ Every neuron within the complex necessarily shapes the probability of possible past states (causes) and future states (effects) of the complex, depending on how it is connected to the other neurons and on its state (say firing strongly for 100 ms). Thus, a neuron firing strongly in a certain visual area may specify as more likely those past states of the complex that are compatible with the invariant concept ‘J.A.'s face’, as well as certain appropriate future states. Another neuron firing strongly in another visual area may specify that there likely was a horizontal edge in a certain position of the visual field, and so on. Yet other neurons that are part of the complex but are silent may specify that certain past (and future) states are unlikely to have occurred (or to occur), such as those having to do with the invariant concepts ‘book’, ‘square’ and so on. Moreover, combinations of neurons may specify higher order concepts, such as ‘J.A. with a red hat sitting on the couch on the left’. Note that all the concepts are specified by elements of the complex, specify cause–effect repertoires over elements of the complex, and acquire meaning intrinsically, in relation to the other concepts in the quale, and not by referring to external inputs (J.A. is just as meaningful when daydreaming about her, or in a dream) [80].

In principle, then, the postulates of IIT offer a way to analyse any system of mechanisms in a particular state and determine whether it constitutes a complex, over which spatial and temporal grain,^11^ and which quale it specifies. Furthermore, while in practice it is not possible to determine the quale and *Φ*^max^ precisely for a realistic system, it is already possible to employ IIT for prediction, explanation and extrapolation.

### (d) Predictions

A straightforward experimental prediction of IIT is that the loss and recovery of consciousness should be associated with the breakdown and recovery of the brain's capacity for information integration. This prediction has been confirmed using transcranial magnetic stimulation (TMS) in combination with high-density EEG in conditions characterized by loss of consciousness [95,96]. These include deep sleep, general anaesthesia obtained with several different agents and brain-damaged patients (vegetative, minimally conscious, emerging from minimal consciousness, locked-in). If a subject is conscious when the cerebral cortex is probed with a pulse of current induced by the TMS coil from outside the skull, the cortex responds with a complex pattern of reverberating activations and deactivations that is both widespread (integrated) and differentiated in time and space (information rich) [95]. By contrast, when consciousness fades, the response of the cortex becomes local (loss of integration) or global but stereotypical (loss of information). The *perturbational complexity index* (PCI), a scalar measure of the compressibility of the EEG response to TMS inspired by IIT, decreases distinctly in all the different conditions of loss of consciousness and, critical for a clinically useful device, is high instead in each conscious healthy subject or neurological patient tested so far [96].

A theory is the more powerful the more it makes correct predictions that violate prior expectations. One counterintuitive prediction of IIT is that a system such as the cerebral cortex may generate experience even if the majority of its pyramidal neurons are nearly silent, a state that is perhaps approximated through certain meditative practices that aim at reaching ‘naked’ awareness without content [97,98]. This corollary of IIT contrasts with the common assumption that neurons only contribute to consciousness if they are active in such a way that they ‘signal’ or ‘broadcast’ the information they represent and ‘ignite’ fronto-parietal networks [3]. That silent neurons can contribute to consciousness is because, in IIT, information is not in the message that is broadcast by an element, but in the form of the conceptual structure that is specified by a complex. Inactive elements of a complex specify a cause–effect repertoire (the probability of possible past and future states) just as much as active ones (think of the dog that did not bark in the famous Sherlock Holmes story). Conversely, if the same neurons were not merely inactive, but pharmacologically or optogenetically inactivated, they would cease to contribute to consciousness: even though their actual state is the same, they would not specify a cause–effect repertoire, since they do not affect the probability of possible past and future states of the complex.^12^

Another counterintuitive prediction of IIT is that if the efficacy of the 200 million callosal fibres through which the two cerebral hemispheres communicate with each other were reduced progressively, there would be a moment at which, for a minimal change in the traffic of neural impulses across the callosum, there would be an all-or-none change in consciousness: experience would go from being a single one to suddenly splitting into two separate experiencing minds (one linguistically dominant), as we know to be the case with split-brain patients [101,102]. This would be the point at which *Φ*^max^ for the whole brain would fall below the value of *Φ*^max^ for the left and for the right hemisphere taken by themselves.

More generally, IIT predicts that, whatever the neural correlate of consciousness (NCC) turns out to be—whether it is global or local within the cortex, anterior or posterior, medial or lateral, whether it includes primary areas or not, the thalamus or not, whether it encompasses neurons in supragranular, infragranular layers of cortex or not—it should be a local maximum of *Φ*, and thus of a maximum of intrinsic, irreducible cause–effect power. IIT also predicts that the NCC is not necessarily fixed, but may expand, shrink and even move within a given brain depending on various conditions. In fact, there may even be multiple NCCs in a single brain, as shown by split-brain patients, in which case there should be multiple local maxima of integrated information. Finally, IIT makes precise predictions about the physical elements that constitute the NCC and the time intervals and levels of activity at which they operate [77,79]: they should have a spatial scale that achieves the highest value of *Φ*, as opposed to finer or coarser grains (say, either individual neurons or local groups of neurons rather than neuronal compartments or brain areas); they should operate most effectively (highest value of *Φ*) at the time scale of consciousness, as opposed to finer or coarser scales (say, hundred milliseconds rather than a millisecond or ten seconds); and the activity states that make the most difference to the NCC should be the ones that support phenomenological distinctions (say, bursting, high mean firing, low mean firing). In short, the general rule is that the NCC must always correspond to a maximum of intrinsic, ireducible cause–effect power.

### (e) Explanations

IIT offers a coherent, principled account of the NCC—which it identifies with the major complex in a particular state—and of many disparate empirical observations. For example, why is consciousness generated by the cerebral cortex (or at least some parts of it), but the cerebellum does not contribute to it, despite the latter having even more neurons; [103]? Why does consciousness fade early in sleep, although the brain remains active? Why is it lost during generalized seizures, when neural activity is intense and synchronous? Why is there no direct contribution to consciousness from neural activity within sensory pathways (the retina) and motor pathways (the motoneurons in the spinal cord), or within neural circuits looping out of the cortex into subcortical structures and back, despite their manifest ability to influence the content of experience?

These and other well-known facts find a parsimonious explanation based on the postulates of IIT. Thus, a prominent feature of the cerebral cortex, which is responsible for the content of consciousness, is that it is composed of elements that are functionally specialized and at the same time can interact rapidly and effectively. This is the kind of organization that yields a comparatively high value of *Φ*^max^. Instead, the cerebellum is composed of small modules that process inputs and produce outputs largely independent of each other [104,105]. Simulations also show that input and output pathways, while capable of affecting the major complex and being affected by it, can remain excluded from it, because they are not part of a local maximum of integrated information. The same applies to loops that may exit the major complex and reenter it. Other simulations show that *Φ*^max^ is low when the effective connectivity among a set of elements is weak or is organized in homogeneous manner. Indeed, as was mentioned above, when consciousness fades during deep slow wave sleep or in certain states of general anaesthesia, the interactions among different cortical regions become weaker or highly stereotypical, as they do during generalized epileptic seizures.

### (f) Extrapolations

Finally, the more the postulates of IIT are validated in situations in which we are reasonably confident about whether and how consciousness changes, the more we can use the theory to extrapolate and make inferences about situations where we are less confident—brain-damaged patients, newborn babies, alien animals, complicated machines and other far-fetched scenarios, as we shall consider next.

## 5. Everywhere?

In the ‘Canticle of the Creatures’, Saint Francis addressed animals, flowers and even stones as if endowed with soul, and praised them as mother earth, brother sun, sister moon, the stars, the air, water and fire. And he was not alone. Some of the brightest minds in the West embraced some form of the ancient philosophical doctrine of panpsychism, starting with the Presocratics and Plato. The Renaissance philosophers Patrizi, Bruno, Telesio and Campanella took the position that matter and soul are one substance. Later, Spinoza, Leibniz, Schopenhauer and, closer to modern times, James, Whitehead, Russell, and Teilhard de Chardin espoused panpsychist notions [106,107]. Strawson [108,109] is a well-known contemporary defender of panpsychism. Eastern traditions, such as Buddhism, have always emphasized the continuity of consciousness across life.

Materialism, or its modern offspring, physicalism, has profited immensely from Galileo's pragmatic stance of removing subjectivity (mind) from nature in order to describe and understand it objectively—from the extrinsic perspective of a manipulator/observer. But it has done so at the cost of ignoring the central aspect of reality from the intrinsic perspective—experience itself. Unlike idealism, which does away with the physical world, or dualism, which accepts both in an uneasy marriage, panpsychism is elegantly unitary: there is only one substance, all the way up from the smallest entities to human consciousness and maybe to the World Soul (*anima mundi*). But panpsychism's beauty has been singularly barren. Besides claiming that matter and mind are one thing, it has little constructive to say and offers no positive laws explaining how the mind is organized and works.

IIT was not developed with panpsychism in mind (*sic*). However, in line with the central intuitions of panpsychism, IIT treats consciousness as an intrinsic, fundamental property of reality. IIT also implies that consciousness is graded, that it is likely widespread among animals, and that it can be found in small amounts even in certain simple systems. Unlike panpsychism, however, IIT clearly implies that not everything is conscious. Moreover, IIT offers a solution to several of the conceptual obstacles that panpsychists never properly resolved, like the problem of aggregates (or combination problem [107,110]) and can account for its quality. It also explains why consciousness can be adaptive, suggesting a reason for its evolution.

### (a) Consciousness is a fundamental property

The axioms and postulates of IIT say that consciousness is a fundamental, observer-independent property that can be accounted for by the intrinsic cause–effect power of certain mechanisms in a state—how they give form to the space of possibilities in their past and their future. An analogy is mass, which can be defined by how it curves space–time around it—except that in the case of experience the entities having the property are not elementary particles but complexes of elements, and experience comes not in two but in a trillion varieties. In this general sense, at least, IIT is not at odds with panpsychism.

### (b) Consciousness comes in various qualities

Unfortunately, panpsychism is mute when it comes to explaining the way any one conscious experience feels—why the perception of red feels different from that of blue and why colours are experienced as different from tones. Instead, at least in principle, IIT says exactly what determines the quality of an experience—what makes it the particular way it is: an experience is a maximally irreducible conceptual structure or quale—a shape in a fantastically high-dimensional cause–effect space specified by a complex of neurons in a particular state. This is the constellation of concepts through which the neurons of the major complex, in various combinations, give form to the space of its possible past and future states (figure 4). Different experiences—every different scene in a movie or in a dream—correspond to different shapes, with some shapes being measurably closer (red and blue) and some more distant within the space (a black screen and a city scene). Indeed, there is much scope for future research to begin mapping psychophysics, for example, the circular nature of colour space, onto the geometry of shapes in cause–effect space—except that a shape in cause–effect space, unlike the shape of an object in 3D space, is the shape within, the shape of experience itself. It is the voice in the head, the light inside the skull.

### (c) Consciousness is adaptive

IIT takes no position on the function of experience as such—similar to physics not having anything to say about the function of mass or charge. However, by identifying consciousness with integrated information, IIT can account for why it evolved, another aspect about which panpsychism has nothing to say. In general, a brain having a high capacity for information integration will better match an environment with a complex causal structure varying across multiple time scales, than a network made of many modules that are informationally encapsulated. Indeed, artificial life simulations (‘animats’) of simple Braitenberg-like vehicles that have to traverse mazes and whose brains evolve by natural selection over 60 000 generations show a monotonic relationship between (simulated) integrated information and adaptation [111,112]. That is, the more adapted individual animats are to their environment, the higher the integrated information of the major complex in their brain. Similar animats, evolved to catch falling blocks in a *Tetris*-like scenario, demonstrate that increased adaptation leads to increased number of concepts in the major complex and an associated increase in integrated information that depends on the complexity of the animats’ environment [113]. Thus, evolution by natural selection gives rise to organisms with high *Φ*^max^ because, given constraints on the number of elements and connections, they can pack more functions per element than their less integrated competitors and thus are more adept at exploiting regularities in a rich environment.

### (d) Consciousness is graded

**Figure 5.**
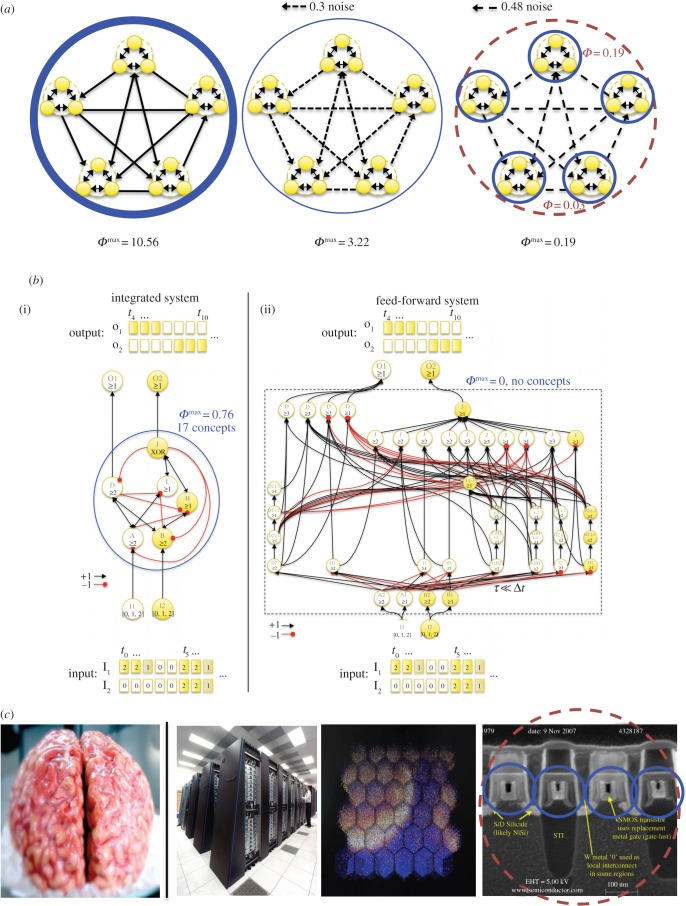
IIT makes several predictions about which systems can experience anything—how much and in which way—and which systems, even complicated ones, have no experience, remaining ‘in the dark’. IIT implies that consciousness is graded (*a*); that aggregates are not conscious (*a*, right panel); that strictly feed-forward systems are not conscious (*b*, right panel), even if they are functionally equivalent in terms of their input–output operations to feedback networks that are conscious (*b*, left panel); that even accurate biophysical simulations of the human brain running on digital machines would not be conscious like us, but would be mere aggregates of much simpler systems (transistors and the like) having minimal *Φ*^max^ (*c*). The last row (*c*) shows, from left to right, a human brain (Allen Institute), the IBM Blue Gene P supercomputer, a columnar model of mouse cortex (Blue Brain Project) and a scanning electron micrographic cross-section of 4 NMOS INTEL transistors in a grid.

IIT does side with the panpsychist intuition that consciousness may be present across the animal kingdom, and even beyond, but in varying degrees. Everything else being equal, integrated information, and with it the richness of experience, is likely to increase as the number of neurons and the abundance of their interconnections grow, although sheer number of neurons is not a guarantee, as shown by the cerebellum. It is also likely that consciousness is graded across the lifetime of any one organism. In us it becomes richer as we grow from a baby to an adult whose brain has fully matured and becomes more functionally specialized. It can also wax and wane when we are highly alert or drowsy, intoxicated by drugs or alcohol, or become demented in old age. This is illustrated schematically in figure 5*a*, where a set of ‘cortical’ areas is integrated into a major complex of ‘high’ *Φ*^max^ when the inter-areal connections are strong, undergoes a reduction in *Φ*^max^ when connection strength is reduced by neuromodulatory changes (simulated as an increase in noise), and finally breaks down into small complexes of low *Φ*^max^.

A corollary of IIT that violates common intuitions is that even circuits as simple as a ‘photodiode’ made up of a sensor and a memory element can have a modicum of experience [80] (see also figure 5*a*, right panel). It is nearly impossible to imagine what it would ‘feel like’ to be such a circuit, for which the only phenomenal distinction would be between ‘this rather than not this’ (unlike a photodiode, when we are conscious of ‘light’ or of ‘dark,’ our experience is what it is because it includes scores of negative concepts, such as no colours, no shapes, no thoughts and so on, that are all available to us). But consider that normal matter at −272.15°C, one degree above absolute zero, still contains some heat. However, in practice its temperature is as cold as it gets. Similarly, there may well be a practical threshold for *Φ*^max^ below which people do not report feeling much of anything, but this does not mean that consciousness has reached its absolute minimum, zero. Indeed, when we fall into a deep, dreamless sleep and don't report any experience upon being awoken, some small complex of neurons within our sleeping brain will likely have a *Φ*^max^ value greater than zero, yet that may not amount to much compared to that of our rich, everyday experience.

### (e) Multiple consciousnesses

IIT also allows for the possibility of two or more complexes coexisting within a single system [80]. Depending on the exact connectivity, these are likely to have quite different values of *Φ*^max^. Indeed, in the brains of both vertebrates and invertebrates, there may well exist, at least under some conditions, a major complex and one or more minor complexes. In humans, the complex that supports our day-to-day stream of conscious experience should have by far the highest value of integrated information—it should be the *major* complex. In split-brain patients the speaking, major complex is unaware of the presence of another consciousness, one that typically lacks speech, but which can be revealed by clever experimental paradigms [102,114]. It is conceivable that at least some cases of ‘high-level’ performance found in normal subjects [64,115]), while unconscious from the perspective of the major complex, may be due to the presence of minor complexes (of course, some of these behaviours may be mediated by purely feed-forward circuits). This counterintuitive scenario of ‘many conscious minds within a single brain’ could be assessed, at least in principle, by measurements of integrated information at the neuronal level. Major and minor complexes may also occur in patients with Marchiafava–Bignami disease [116] and other disconnection syndromes, in patients with identity and conversion disorders [63], and in other neurological and psychiatric conditions.

### (f) Aggregates are not conscious

‘Take a sentence of a dozen words, and take twelve men and tell to each one word. Then stand the men in a row or jam them in a bunch, and let each think of his word as intently as he will; nowhere will there be a consciousness of the whole sentence’. This is how William James illustrated the combination problem of panpsychism [110]. Or take John Searle: ‘Consciousness cannot spread over the universe like a thin veneer of jam; there has to be a point where my consciousness ends and yours begins’ [117]. Indeed, if consciousness is everywhere, why should it not animate the United States of America? IIT deals squarely with this problem by stating that only maxima of integrated information exist. Consider two people talking: within each brain, there will be a major complex—a set of neurons that form a maximally irreducible cause–effect structure with definite borders and a high value of *Φ*^max^. Now let the two speak together. They will now form a system that is also irreducible (*Φ* > zero) due to their interactions. However, it is not maximally irreducible, since its value of integrated information will be much less than that of each of the two major complexes it contains. According to IIT, there should indeed be two separate experiences, but no superordinate conscious entity that is the union of the two. In other words, there is nothing-it-is-like-to-be two people, let alone the 300 plus million citizens making up the USA.^13^ Again, this point can be exemplified schematically by the system of figure 5*a*, right panel. While the five small complexes do interact, forming a larger integrated system, the larger system is not a complex: by the exclusion postulate, only the five smaller complexes exist, since they are local maxima of integrated information (*Φ*^max^ = 0.19), while the larger system is not a complex (*Φ* = 0.03). Worse, a dumb thing with hardly any intrinsically distinguishable states, say a grain of sand for the sake of the argument, has no experience whatsoever. And heaping a large number of such zero-*Φ* systems on top of each other would not increase their *Φ* to a non-zero value: to be a sand dune does not feel like anything either—aggregates have no consciousness.

### (g) Complicated systems can be unconscious

A second class of zero-*Φ* systems are purely feed-forward computational networks in which one layer feeds the next one without any recurrent connections. In a feed-forward network, the input layer is always determined entirely by external inputs and the output layer does not affect the rest of the system, hence neither layer can be part of a complex, and the same is true recursively for the next layers downstream and upstream. According to IIT, then, a feed-forward network does not exist intrinsically—for itself—but is a zombie—carrying out tasks unconsciously [118]. Yet from the extrinsic perspective of a user, feed-forward networks, like those used in deep learning, perform plenty of useful computational functions, such as finding faces or cats in images [119], labelling images, reading zip codes and detecting credit card fraud.

This has a rather startling consequence. Consider that any neural network with feedback circuits can be mapped onto a purely feed-forward network in such a manner that the latter approximates its input–output relationships (for computations bounded by a maximal time step [120]). That is, for the same inputs, the two networks will yield the same output (in general, the equivalent feed-forward network will have many more nodes and connection than the feedback network). Therefore, a purely feed-forward system able to replicate the input–output behaviour of the human brain (under the limited time-step constraint) would be behaviourally indistinguishable from us, and certainly capable of passing the Turing test, yet it would have zero *Φ* and would thus be a ‘perfect’ zombie. A simple example of two functionally equivalent systems, one with recurrent connections and non-zero *Φ*, and one purely feed-forward with zero *Φ*, is shown in figure 5*b* [80].

In people and organisms that evolved through natural selection, input–output behaviour provides a good first guess about the presence of consciousness. However, as demonstrated by the example in figure 5*b*, this may not always be the case for radically different computational architectures. In the general case, and certainly with machines, it becomes essential to consider the internal circuitry—not just what the machine does, but how it does it. This also means that there cannot be an ultimate Turing test for consciousness (although, there may be some practical CAPTCHA-like tests [121]). According to many functionalist notions [122], if a machine reproduces our input–output behaviour in every circumstance, it would have to be granted consciousness just as much as us. IIT could not disagree more—no Turing test (e.g. Samantha in the Hollywood movie *She*) can be a sufficient criterion for consciousness, human or otherwise.

### (h) Simulations of conscious neural systems can be unconscious

Finally, what about a computer whose software simulates in detail not just our behaviour, but even the biophysics of neurons, synapses and so on, of the relevant portion of the human brain [123]? Could such a digital simulacrum ever be conscious? Functionalism again would say yes, even more forcefully. For in this case *all* the relevant functional roles within our brain, not just our input–output behaviour, would have been replicated faithfully. Why should we not grant to this simulacrum the same consciousness we grant to a fellow human? According to IIT, however, this would not be justified, for the simple reason that the brain is real, but a simulation of a brain is virtual. For IIT, consciousness is a fundamental property of certain physical systems, one that requires having *real* cause–effect power, specifically the power of shaping the space of possible past and future states in a way that is maximally irreducible intrinsically. In the same way, mass is an intrinsic property of systems of particles, a property that has real causal power, specifically that of bending space–time. Therefore, just like a computer simulation of a giant star will not bend space–time around the machine, a simulation of our conscious brain will not have consciousness.^14^ Of course, the physical computer that is running the simulation is just as real as the brain. However, according to the principles of IIT, one should analyse its real physical components—identify elements, say transistors, define their cause–effect repertoires, find concepts, complexes and determine the spatio-temporal scale at which *Φ* reaches a maximum. In that case, we suspect that the computer would likely *not* form a large complex of high *Φ*^max^, but break down into many mini-complexes of low *Φ*^max^. This is due to the small fan-in and fan-out of digital circuitry (figure 5*c*), which is likely to yield maximum cause–effect power at the fast temporal scale of the computer clock.^15^

## 6. Conclusion

In summary, there are some aspects of IIT that definitely do not fit with panpsychism, and others that vindicate some of its intuitions. In this respect, it is natural to consider how one should regard some of the inferences derived from IIT for which it is hard even to imagine a direct test at the present time. Our position is that, as is often the case in science,^16^ a theory is first tested and validated in situations that are close to ideal, and then extrapolated to more remote cases. Ideally, whether consciousness varies with integrated information, and other predictions of IIT, would first be validated *here*—on my own consciousness: for example, does *Φ*^max^ collapse when I undergo general anaesthesia or a seizure, or when I fall into dreamless sleep, and return to high values when I dream? Does my experience change if one temporarily inactivates a region of my brain that is part of the major complex, but not one that is outside it? Does it change if one succeeds in connecting a neuromorphic microcircuit that becomes part of my major complex and not otherwise? Then one can extrapolate to *there*, at first in situations involving other healthy humans, then in slightly more difficult cases, say monkeys with a brain similar to ours who are trained to give reports similar to ours. Finally, insofar as the theory has been validated and has shown good predictive and explanatory power, one can try and extrapolate to *everywhere*, unresponsive patients with just a small ‘island’ of functioning brain tissue, newborn babies, animals very different from us, photodiodes, machines, and computer simulations. After all, often in science the most we can do is to draw our best inferences about unknown instances based on a theory that works well in many known instances. And that is much better than to make arbitrary claims or to draw no inference whatsoever.

## Endnotes

1 Note that we consider reflective–consciousness, highly developed in adult humans, to be a subclass of conscious experiences. Likewise, the feeling of freely willing an action—such as raising one's arm—sometimes also referred to as *agency* [6,7]—is another subclass of conscious experiences. While their content differs from the content associated with feeling pain or seeing red, subjectivity is common to all.

2 Consciousness can be dissociated from many other cognitive processes that have traditionally been closely linked to it, including memory, emotions and selective attention (for reviews see [19,20]). It can persist if the recall of long-term memories is impaired, it can be present in patients who lack affect, and it can be dissociated from attention. The last point is particularly counterintuitive but is well supported—subjects can attend to invisible objects [21]. The extent to which it is possible to become conscious of something without also attending to it is more controversial [21,22].

3 Not to mention the question of whether it feels-like-something to be a Venus flytrap or a single-cell organism.

4 If it is not outright wrong, IIT most likely will have to be refined, expanded and adjusted. However, in its current form (IIT 3.0), it explains and predicts a wide range of phenomena, including a number of counterintuitive predictions amenable to empirical falsification. For the latest formulation of the theory, see [80]; for earlier versions, see [76,77,81,82]; for a literary account, see [77,83]. The main differences between IIT 3.0 and earlier versions are listed in the appendix of [80].

5 For instance, the unified nature of conscious experiences has been questioned by psychophysical experiments demonstrating temporal asynchrony [85,86]. See also [87].

6 For example, the notion of the aether was introduced in the late nineteenth century to explain the propagation of light. When more and more experiments concluded that, whatever the aether might be, it had no effects whatsoever, it finally fell under Occam's razor, and it plays no role in modern physics.

7 Importantly, this may be a macro- rather than a micro-spatio-temporal scale [79]. For example, the relevant level for human consciousness is likely to be neurons at the scale of 100 ms, rather than molecules at the nanosecond scale. Note that it is possible for a single physical system, such as the brain, to contain two or more independent yet causally interacting complexes, each with their own *Φ*^max^ (see section on multiple consciousnesses). Indeed, it is even possible for a physical system to contain complexes at different spatio-temporal grains, such as a mitochondrion forming a complex inside a neuron, as long as there is no causal overlap at the relevant scales.

8 Requiring that only the maximum of *Φ* over elements, spatial and temporal grain must be considered is not exceptional in science: many of the laws of physics are formulated as extremum principles, e.g. the principle of least action.

9 IIT postulates that experience is a fundamental, intrinsic property of the world. Different experiences must be specified by different physical substrates, although different physical substrates may specify the same experience (for example, by differing in micro-properties that are causally irrelevant at the macro-scale that achieves a maximum of cause–effect power, see also *metamers*) [89]. Note that IIT is compatible with quantum mechanics. In principle, *Φ* and related quantities can be assessed also in quantum system, although it has been suggested that at the quantum level *Φ* values may be very small [90].

10 Here we do not elaborate about particular cortical areas, cortical layers or particular population of neurons.

11 The exclusion postulate requires that the set of mechanisms that specify one particular experience do so over the time window at which *Φ* reaches a maximum. If the next experience involves an overlapping set of mechanisms, it would seem that, to avoid multiple causation, it should be specified over a non-overlapping time window. Accordingly, the seemingly continuous ‘stream’ of consciousness would actually be constituted by a discrete succession of ‘snapshots’, in line with some psychophysical evidence [91–94]. Note that each snapshot has motion and other dynamic percepts associated with it.

12 It is instructive to consider ‘the perfect experiment’ hypothesized by Cohen & Dennett [99]: a subject looks at a red apple, and neurons in his cortical colour region (say V4) fire. However, imagine one could selectively block their projections to further cortical regions, so that the subject cannot access and report the colour of the apple. According to Cohen and Dennett, any theory claiming that ‘phenomenal’ consciousness can be dissociated from cognitive access would have to claim that, as long as the colour neurons are active, the subject would be conscious of a red apple, while at the same time he would be denying that it is red. According to IIT, however, blocking the projections of V4 neurons destroys their cause–effect repertoires no matter whether they are firing or not, leading to the collapse of the ‘colour section’ (Q-fold) of the conceptual structure that corresponds to the subject's experience. As a consequence, the subject would not be conscious of colours (*cerebral achromatopsia*) and would not even understand what he has lost (*colour anosognosia*), just like a patient described by von Arx *et al.* [100].

13 By the same token, the exclusion postulate predicts a scenario that is the mirror image of the prediction that consciousness will suddenly split in two when the corpus callosum is ‘cooled’ below a critical point: if two people speaking were to increase their effective causal interactions by some, yet to be invented, direct brain-to-brain connectivity booster, to the point where the *Φ*^max^ of the two interacting brains would exceed *Φ*^max^ of the individual brains, their individual conscious mind would disappear and its place would be taken by a new Über-mind that subsumes both.

14 A similar point was made by John Searle with his Chinese Room Argument [124] and by Leibniz 300 years earlier with his mill [125].

15 Ultimately, any digital computer running software can be mimicked by a Turing Machine with a large state-transition matrix, a moving head that writes and erases, and a very, very long memory tape—in that case, cause–effect power would reside in the moving head that follows one out of a few instructions at a time. On the other hand, there is no reason why hardware-level, neuromorphic models of the human brain that do not rely on software running on a digital computer, could not approximate, one day, our level of consciousness [126]. A related question has to do with the Internet and whether it could be conscious [127]. One way to think about this is to assume that each computer connected to the Internet is an element having real cause–effect power at a macro-level (by ‘black-boxing’ its internal mechanisms). For example, each computer could send an ON signal when it is ON and an OFF signal when it is OFF. One could then make sure that each computer increased or decreased the likelihood of being ON depending on how many ON signals it receives. In principle, this kind of organization could be arranged so that it gives rise to a complex of high *Φ*, although this is certainly not the way the Internet works right now. On the other hand, if one considers the micro-elements inside each computer (say its transistors) as having real cause–effect power, we are back to the situation in which they most likely would not form any large complex within each computer, let alone across connected computers.

16 A well-known instance of such an extrapolation is the inference of singularities in space–time due to the extreme mass of a stellar object. Such black holes were pure conjectures, based on a solution of Einstein's theory of General Relativity, until they were subsequently confirmed observationally.
